# Unmixing for ultra-high-plex fluorescence imaging

**DOI:** 10.1038/s41467-022-31110-z

**Published:** 2022-06-16

**Authors:** Archibald Enninful, Alev Baysoy, Rong Fan

**Affiliations:** 1 Department of Biomedical Engineering, Yale University, New Haven, CT 06520 USA; 2 Department of Pathology, Yale School of Medicine, New Haven, CT 06520 USA; 3 Yale Cancer Center and Yale Stem Cell Center, Yale School of Medicine, New Haven, CT 06520 USA; 4 Human and Translational Immunology Program, Yale School of Medicine, New Haven, CT 06520 USA

**Keywords:** Imaging, Proteomics

## Abstract

Writing in Nature communications, Seo and collaborators presented PICASSO as a method to achieve 15-color imaging of spatially overlapping proteins using no reference emission spectra in a single staining and imaging round. This accessible tool has the potential to be applied to diverse applications within the spatial biology field without neglecting accuracy.

## Mixing it up

Multiplexed biomolecular imaging of heterogeneous tissues is essential for a variety of biological and biomedical research. In particular, high-plex immunofluorescence imaging such as CODEX^1^, CyCIF^2^, 4i^3^, immunoSABER^4^, and NanoString CosMx^5^ can detect tens of protein markers to identify heterogeneous cell types and cell-cell interactions at cellular or even subcellular resolution, enabling the study of architectural and spatial relationships of tissue in situ. Central to most of these approaches is the cyclic imaging which is time consuming and may result in signal loss due to photobleaching or possible loss of tissue morphology during the washing and/or quenching steps. Although it has been demonstrated to achieve much higher plex protein imaging using metal isotope labeling for mass spectrometry^6,7^ or organic dyes for Raman microscopy^8^, these techniques require complex antibody tagging and different imaging modalities that are not readily accessible.

When spectrally overlapping fluorophores are used to label biomolecules such as proteins for multicolor fluorescence imaging, it may become difficult to distinguish real signals from false positive signals due to bleed-through. Thus, techniques to accurately and efficiently tell apart the signal from each fluorophore become indispensable. Researchers had developed approaches to mitigate signal noise and overlap in multiplexed imaging studies. The signal in each channel is modeled as a linear combination of the contributing fluorophores^9^. By employing a mixing matrix, linear unmixing can produce an unmixed image. The major drawback with the linear unmixing approach is that a reference spectrum is needed. For example, in a multiplexed immunofluorescence experiment with two or three fluorophores that have overlapping emission spectra, to quantify the signal unique to each fluorophore, a reference, which could be a region of the tissue section of interest with ‘pure’ fluorophore can be used. This can be problematic in highly heterogeneous tissues such as the brain. Recently developed algorithms like LUMoS employ unsupervised machine learning approaches to identify the spectral signatures unique to each fluorophore in a method termed as *blind unmixing* precludes the need to have a reference spectrum^10^. However, challenges still remain with this approach making higher-level multiplexing difficult.

## PICASSO masters yet another art

**Fig. 1:**
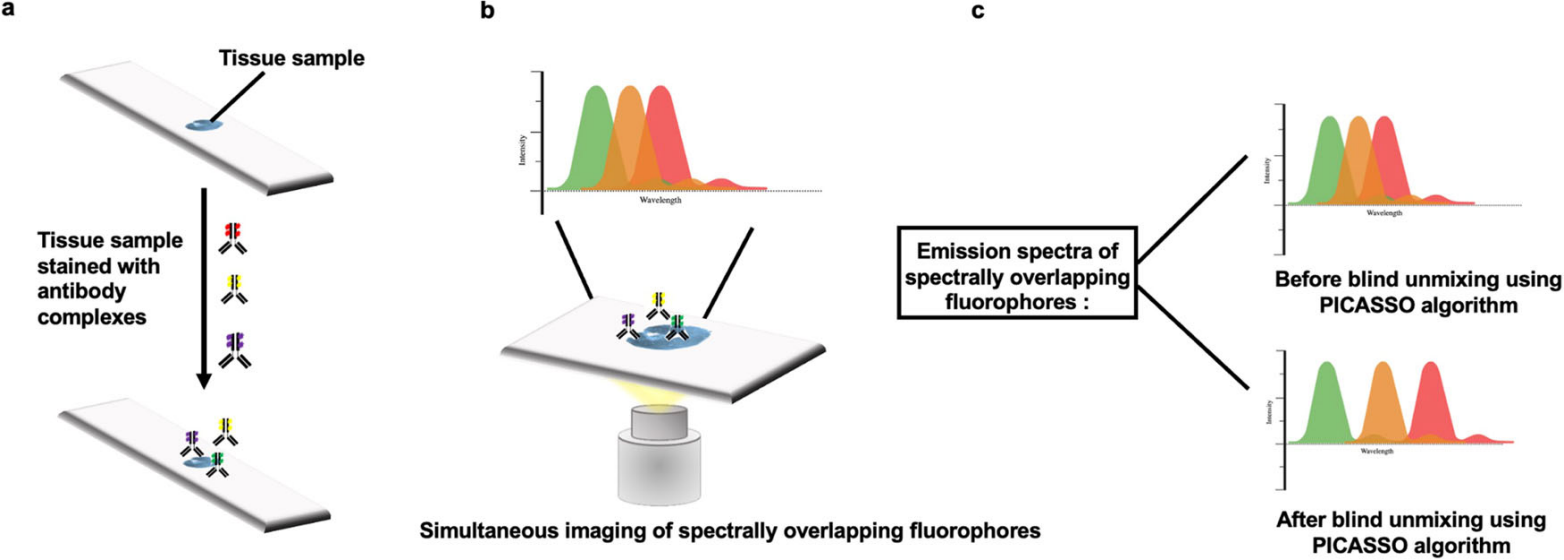
Working Principle of PICASSO. **a** Simultaneous staining of protein targets using a cocktail of antibodies labeled with fluorophores that have overlapping emission spectra. **b** Single-round imaging to detect targets. **c** Reference-free unmixing via mutual information minimization to construct ultra-high-plex multi-color fluorescence image.

Recently, Seo and collaborators developed PICASSO^11^ (Process of ultra-multiplexed Imaging of biomolecules via the unmixing of the signals of spectrally overlapping fluorophores), an algorithm that can efficiently unmix images without the need for a reference emission spectrum (Fig. 1) the principle of linear unmixing has been previously applied to images composed of signals from spectrally overlapping fluorophores. Given the ample number of targets and the ultra-specificity of techniques like immunofluorescence, better algorithms that allow for strong multiplexing capabilities are necessary. Unlike previously described linear unmixing algorithms, PICASSO works iteratively by minimizing the shared information between mixed images in a pairwise and sequential manner^12^. This approach sidesteps the need for a reference spectrum and generates images which are almost indistinguishable from the “ground truth” images. The PICASSO algorithm developed by Seo and co-authors addresses these challenges by blindly unmixing images without a reference emission spectrum, all the while allowing for greater spectral distinction, to achieve a 45-color multiplexed imaging of the mouse brain in only three rounds of staining and imaging. PICASSO works by iteratively reducing the mutual information between mixed images. Applying principles from information theory, the authors designed the algorithm to take as input, the same number of fluorophores and mixed images and iteratively minimize the information between them. Central to its workflow is the assumption that due to spectral mixing, mixed images share a significant amount of mutual information, thus by minimizing this information, an unmixed image can be obtained.

To test PICASSO, the authors used two spectrally overlapping fluorophores and two specific detection channels. The authors then used the first detecting channel to detect signal from only the first fluorophore and the second channel detected the signal from both fluorophores. Representing this information as a matrix, the authors can calculate a parameter, α, which is the ratio of the fluorescence intensity of the first fluorophore in the second channel to the fluorescence intensity of the first fluorophore in the first detection channel. By subtracting the second image from the first image (after scaling by α), the ground truth image of the second channel can be obtained. This elegant and straightforward algorithm is then scaled up for higher-level multiplexing using multiple fluorophores and detection channels. As part of the workflow, PICASSO allows for the simultaneous imaging of a large number of proteins, only constrained by the number of fluorophores available for each of the excitation lasers to be used in that specific experiment. The authors used primary antibody-Fab complexes which allow for primary antibodies to be non-covalently linked to reporter molecules, which in this case is the Fab fragment of a secondary antibody conjugated to a fluorophore^13^. In essence, the primary antibodies used can all be raised in the same host species, since the primary antibodies are assembled as a complex with the secondary antibody with a fluorophore. Antibodies were validated to ensure minimal crosstalk between one another and to ensure accurate staining patterns consistent with literature reports.

PICASSO performed significantly better than linear unmixing approaches. Using four overlapping fluorophores (CF488A, ATTO488, ATTO514, and ATTO532), all excited using the 488-nm laser on a mouse brain section, the authors obtained images for NeuN from five brain regions: CA1, CA3, cortex, dentate gyrus, and thalamus. These images were much closer to the ground truth images taken with a single fluorophore as compared with images from linear unmixing methods. PICASSO significantly outperformed its peers, registering structural similarity (SSIM) >99%. PICASSO can also unmix spatially overlapping proteins effortlessly. The algorithm was able to unmix images from spatially overlapping proteins: PV, NeuN and GFAP in a mouse brain section. Interestingly, PICASSO showed a robust performance, and is agnostic to the ratio of protein expression levels which can vary in a tissue region or in brightness of the fluorophores. Next, it was demonstrated that the efficacy of their technology by using PICASSO with multiple excitation lasers by performing a 15-color multiplexed imaging of the mouse hippocampus using PICASSO, identifying proteins such as IBA1, GFAP and SOX2. PICASSO was also able to demonstrate spectrally distinct large brain imaging of the dentate gyrus of the mouse hippocampus with multiple excitation lasers. In this experiment, Seo and collaborators were able to demonstrate the consistent cellular organization of the blood brain barrier. Finally, PICASSO was also demonstrated to be capable of multiplexed mRNA FISH, simultaneous imaging of proteins and mRNA after tissue clearing without problems with background autofluorescence. The development of algorithms such as PICASSO reduces the need for repeated staining and imaging processes, reducing experimental time and effort without compromising quality. PICASSO does not need complicated optics and can be readily adopted in most conventional laboratories. Thus, it has the potential to be applied broadly, particularly in the area of spatial multi-omics, improving the throughput of imaging-based approaches such as in-situ hybridization.

## Broader impacts

Extending beyond the basic research applications of PICASSO are its clinical benefits. Through the lens of clinical cancer imaging, as an example, early detection and diagnosis, detection of cancer recurrence, and ability to map the tumor microenvironment at the spatial level are integral components for therapy selection. PICASSO can be implemented to allow for visualization of multiple cell-surface protein targets within a tumor tissue section to detect spatial organization of target proteins and understand the protein-protein interactions and co-localization within the structure of the tissue. For this reason, PICASSO offers to the clinical research field an accurate and robust technology to elucidate potential therapeutic targets in disease models.

PICASSO is a versatile and user-friendly tool that has potential to provide granularity in heterogeneous tissue samples through distinguishing fluorophores. In addition, this technology is able to be combined with other modalities that allow it to be useful for a broad range of applications in which spatial information is critical. One shortcoming of multiplexed imaging is the noise that is introduced into the readouts—fluorescence microscopy is limited by the broad fluorescence spectral width. A possible solution to this limitation could be by combining PICASSO with excitation unmixing which has been validated in other studies^14^.
